# Harnessing the Antimicrobial Potential of Baicalein-Coated Fe_3_O_4_/Ag Nanoparticles for Biomedical and Environmental Applications

**DOI:** 10.1021/acsami.6c02389

**Published:** 2026-04-24

**Authors:** Jeniffer Blair, Garima Rathee, Antonio Puertas-Segura, Kristina Ivanova, Aleksandra Ivanova, Leonardo Martín Pérez, Tzanko Tzanov

**Affiliations:** † Grup de Biotecnologia Molecular i Industrial, Department of Chemical Engineering, Universitat Politècnica de Catalunya (UPC-BarcelonaTech), Rambla Sant Nebridi 22, Terrassa, 08222, Spain; ‡ Cleantech Bulgaria Foundation, 11 Lukashov str., floor 6, Vratsa, 3000, Bulgaria

**Keywords:** antibiotic resistance, magnetic nanoparticles, antibacterial, antibiofilm, quorum sensing, water treatment

## Abstract

The increasing prevalence of antimicrobial resistance (AMR) and biofilm-associated infections represents a major challenge in both biomedical and environmental settings. Herein, we report the synthesis and comprehensive evaluation of a multifunctional nanocomposite composed of baicalein-coated Fe_3_O_4_/Ag nanoparticles (FAB NPs), designed to combine magnetic recoverability, controlled silver (Ag^+^) release, antimicrobial activity, and improved biocompatibility. FAB NPs were synthesized via a stepwise coprecipitation approach and characterized in terms of morphology, structure, surface chemistry, magnetic properties (normalized saturation magnetization of 27.2 ± 0.1 emu/g), antioxidant capacity (43.8% 2,2-diphenyl-1-picrylhydrazyl inhibition at 1.5 mg/mL), and antibacterial potential (minimum inhibitory concentrations of 0.19–0.38 mg/mL), as well as Ag^+^ release behavior. The developed nanocomposite exhibited strong broad-spectrum antibacterial activity against *Staphylococcus aureus*, *Escherichia coli*, and *Pseudomonas aeruginosa*, together with pronounced inhibition of biofilm formation (>60%) and quorum sensing (QS), while achieving a 33-fold reduction in Ag^+^ release compared to nonfunctionalized Fe_3_O_4_/Ag nanoparticles. Mechanistic studies revealed that FAB NPs disrupt bacterial membranes and interfere with QS pathways (suppressing 25% of violacein expression), contributing to their antibiofilm efficacy. Importantly, FAB NPs displayed improved biocompatibility toward human keratinocytes and fibroblasts and did not promote significant resistance development during 55 days of exposure in both Gram-positive and Gram-negative bacteria. As a proof of concept for environmental applications, FAB NPs incorporated into activated carbon packed-bed columns achieved complete removal of the reference faecal indicator *E. coli* from contaminated water, with negligible Ag^+^ leaching. Overall, this work demonstrates that baicalein functionalization enables the design of safe and highly effective Ag-based magnetic nanocomposites, offering a versatile strategy to combat microbial contamination and AMR in both biomedical and water treatment applications.

## Graphical abstract

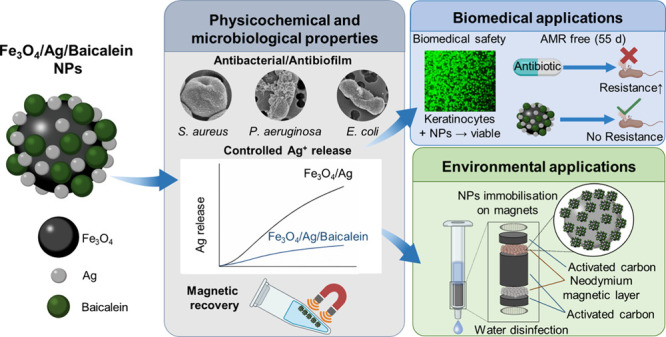

## 1 Introduction

The alarming rise in antimicrobial resistance (AMR), together with the challenges posed by microbial biofilm formation, presents significant concerns in both biomedical and environmental settings. Biofilms not only protect bacterial communities from conventional therapies and disinfection methods but also promote the horizontal transfer of resistance genes, thereby fuelling the spread of multidrug-resistant strains.^1–3^ While these phenomena are most commonly associated with clinical infections, they also pose serious limitations in environmental contexts such as water treatment systems and industrial processes, where biofilms contribute to persistent contamination and the dissemination of resistant microorganisms.^4–6^

Nanotechnology offers promising strategies to combat AMR and microbial biofilm establishment and proliferation, particularly using metal-containing nanoparticles (NPs).^7^ Among these, silver NPs (Ag NPs) have been extensively investigated due to their potent broad-spectrum antimicrobial activity and their ability to disrupt biofilm structures.^8^ Through reduction with an appropriate agent, silver ions (Ag^+^) can be converted into nanoscale particles with applications across diverse sectors, including medicine, textiles, food packaging, and water treatment, among others. However, the practical uses of Ag NPs remain limited by challenges such as aggregation, cytotoxicity, and uncontrolled silver (Ag^+^) release. Hybrid nanomaterials have therefore emerged as a promising strategy to improve NPs stability, control ion release, and enhance overall biological performance.^9^

Magnetite NPs (Fe_3_O_4_ NPs), another widely used class of metal-containing nanomaterials, have also shown promise in a wide range of applications, including biomedicine and wastewater treatment.^10^ In environmental applications, they are particularly attractive as efficient adsorbents due to their magnetic recoverability. When functionalized or integrated into hybrid nanostructures, Fe_3_O_4_-based nanocomposites can contribute to water disinfection, offering safer and more sustainable treatment processes.^11^ This strategy facilitates the postuse recovery of metal-containing NPs, thereby mitigating safety concerns associated with their nonbiodegradability and potential bioaccumulation.^12^ In addition, magnetic supports can stabilize metal-based NPs, limit their dispersion, and enable efficient reuse, potentially reducing operational costs.^13^ Beyond their role in environmental remediation, Fe_3_O_4_ based systems are also widely explored in biomedicine, where stringent criteria related to biocompatibility, inflammatory response, stable metal immobilization, and targeted delivery to specific cells or tissues become critical.^14–16^

Unlike conventional antibiotics, which typically act through a single mechanism,^17,18^ metal-based nanoparticles exert antimicrobial activity through multiple pathways, including membrane disruption, oxidative stress induction, and quorum sensing inhibition,^12,16,19,20^ thereby reducing the likelihood of resistance development.^7^ In parallel, surface functionalization with naturally occurring biomolecules such as baicalein, a flavonoid with antimicrobial, antioxidant, and anti-inflammatory properties, represents an effective strategy to enhance nanoparticle stability, biocompatibility, and therapeutic efficacy while generating synergistic antimicrobial effects.^9,21–28^

In this study, we report the synthesis and characterization of a novel baicalein-coated Fe_3_O_4_/Ag NPs (FAB NPs). By integrating the biocidal activity of Ag^+^, the magnetic responsiveness of Fe_3_O_4_, and the bioactivity of baicalein, we developed a multifunctional nanomaterial with antioxidant, antimicrobial, and biofilm-disrupting properties. FAB also combines magnetic responsiveness, controlled Ag^+^ release, improved biocompatibility, and reduced resistance development against three clinically and environmentally relevant pathogens. Moreover, the applicability of FAB NPs as a filler material for water disinfection was evaluated in packed-bed column systems as a proof of concept. Overall, this work highlights the potential of FAB NPs as a versatile strategy to combat microbial spread and colonisation in both environmental and biomedical contexts.

## 2 Materials and Methods

### 2.1 Reagents and Cells

Iron­(II) chloride (FeCl_2_), iron­(III) chloride hexahydrate (FeCl_3_·6H_2_O), silver nitrate (AgNO_3_), sodium hydroxide (NaOH), baicalein, dimethyl sulfoxide (DMSO), coliform ChromoSelect agar, cetrimide agar, Baird–Parker agar, and Luria–Bertani (LB) medium were purchased from Sigma-Aldrich (Spain). Mueller Hinton broth (MHB) and tryptic soy broth (TSB) were provided by Sharlab (Spain). Phosphate-buffered saline solution (PBS; 100 mM pH 7.4) was obtained from Fisher BioReagents (USA). Phosphatidylethanolamine (PE, No. 840027) and phosphatidylglycerol (PG, No. 841188) extracted from *E. coli* were provided by Avanti Research Inc. (USA). Bacterial strains *Staphylococcus aureus* (ATCC 25923), *Escherichia coli* (ATCC 25922), and *Pseudomonas aeruginosa* (ATCC 10145), human fibroblast cells (ATCC-CRL-4001, BJ-5ta), and human keratinocyte cells (HaCaT cell line) were obtained from the American Type Culture Collection (ATCC LGC Standards, Spain). *Chromobacterium violaceum* (CECT 5999) was purchased from the Spanish Type Culture Collection (CECT, Spain).

### 2.2 Synthesis of Nanoparticles

Fe_3_O_4_, Fe_3_O_4_/Ag, and baicalein-coated Fe_3_O_4_/Ag NPs (hereafter defined as Fe_3_O_4_ NPs, FA NPs, and FAB NPs, respectively) were synthesized using the coprecipitation method, with NaOH acting as a precipitating agent (Figure S1). Synthesis of Fe_3_O_4_ NPs: a solution of Fe^2+^/Fe^3+^ in a molar ratio of 1:2 was prepared by dissolving 50.0 mg of FeCl_2_ and 213.2 mg of FeCl_3_·6H_2_O in 50 mL ultrapure (Milli-Q) water and stirred at 60 °C for 1 h. The pH of the solution was then adjusted to 12 by adding 1 M NaOH under continuous stirring (200 rpm). An immediate color change to black was observed, and the reaction mixture was stirred for an additional hour at 60 °C. The resulting particles were magnetically separated and washed three times with Milli-Q water. Finally, the material was freeze-dried for 24 h using a Telstar LyoAlfa 15 lyophilizer (Italy) and stored at room temperature until further use.

Synthesis of FA NPs: Fe_3_O_4_ NPs were first synthesized as described above. Next, 40 mg of AgNO_3_ was added to the suspension and the mixture was stirred for 30 min at 60 °C. The FA NPs were then washed, lyophilized, and stored as previously described.

Synthesis of FAB NPs: following the synthesis of FA NPs, 25 mg of baicalein (dissolved in 1 mL of DMSO) was added to the NPs suspension and stirred for another 30 min at 60 °C. The resulting particles were magnetically separated and washed three times with Milli-Q water. Finally, FAB were lyophilized and stored until further use as described above.

### 2.3 Characterization of Nanoparticles

The ζ-potential, hydrodynamic size, and polydispersity index (PDI) were measured with a Zetasizer Nano Z (Malvern Instruments Ltd., UK). Prior to analysis, the NPs were dispersed by sonication for 15 min using a Sonopuls HD 2070 ultrasonic homogenizer (Bandelin Electronic GmbH & Co., Germany) with a 3 mm probe. The instrument was operated at 50% amplitude in a pulse mode (0.2 s on/0.8 s off), while the suspension was kept in an ice bath to prevent overheating. X-ray diffraction (XRD) spectra were obtained in a Bruker D8 Advance diffractometer (Bruker Inc., USA). The morphology and the size of the NPs were analyzed using transmission electron microscopy (TEM), high-resolution TEM (HRTEM), scanning TEM (STEM), energy dispersive X-ray (EDX), and selected area electron diffraction (SAED) by casting 10 μL of sample onto a copper grid, air-dried for 15 min, and imaged in a JEOL JEM-2011 high-resolution transmission electron microscope operated at 200 kV (JEOL Ltd., Japan). The NPs were further characterized by X-ray photoelectron spectroscopy (XPS) using a SPECS system equipped with a PHOIBOS 150 MCD-9 XP hemispherical analyzer and a high-intensity XR50 twin-anode X-ray source (Mg Kα, 1253 eV; Al Kα, 1487 eV) operated at 150 W (SPECS Surface Nano Analysis GmbH, Germany). Magnetic properties were measured with a Vibrating Sample Magnetometer (VSM; MicroSense LLC., model ADE–EV9, USA).

Phenolic content was assessed using the Folin–Ciocâlteu spectrophotometric method. Suspensions of Fe_3_O_4_, FA, and FAB NPs were prepared in Milli-Q water (1 mg/mL), after which 20 μL of each sample was mixed with 80 μL of 0.2 N Folin-Ciocâlteu reagent and 100 μL of 20% (w/v) Na_2_CO_3_, and incubated for 10 min in darkness. The absorbance was measured at 765 nm, and the total phenolic content was calculated using a calibration curve prepared with gallic acid as standard.

The antioxidant activity of the Fe_3_O_4_, FA, and FAB NPs was assessed using the 2,2-diphenyl-1-picrylhydrazyl (DPPH) radical scavenging assay. Briefly, 100 μL of NPs suspension was mixed with 300 μL of 60 μM DPPH in methanol and incubated in the dark at room temperature for 30 min. Water and ascorbic acid (1 mg/mL) were used as negative and positive controls, respectively. Absorbance at 517 nm was measured to determine radical scavenging activity.

The content of Ag^+^ in FA and FAB was evaluated by inductively coupled plasma mass spectrometry (ICP-MS; Model 7800, Agilent). Briefly, 25 ± 0.5 mg of NPs were submerged in 1 mL of PBS and incubated at 37 °C and 230 rpm for 7 days. Every 24 h, the suspensions were sampled after magnetic separation using a neodymium magnet at room temperature, and the withdrawn volume was replenished with fresh PBS. Then, each sample was digested in a solution of 20% (v/v) nitric acid at 70 °C for 24 h. The digested samples were diluted with Milli-Q water to adjust the acid concentration of each sample to 2% (v/v) nitric acid. Finally, each sample was filtered using a 0.22 μm Millipore Express PES Membrane filter before quantifying the concentration of Ag^+^ with ICP-MS.

### 2.4 Antibacterial Activity of Nanoparticles

Fe_3_O_4_, FA and FAB NPs were tested against *S. aureus* (Gram-positive), *P. aeruginosa* (Gram-negative) and *E. coli* (Gram-negative) via colony-counting on nutritive agar plates. Bacterial cultures were grown overnight in Mueller-Hinton broth (MHB) at 37 °C with shaking (230 rpm) and subsequently diluted in fresh MHB to an optical density at 600 nm (OD_600_) of approximately 0.01, corresponding to 10^5^–10^6^ colony-forming units per milliliter (CFU/mL). NP dilutions (starting at 3.0 mg/mL) were prepared by 2-fold serial dilution in MHB in 96-well polypropylene microplates. Each well was then inoculated with an equal volume of bacterial suspension (1:1, v/v) and incubated for 24 h at 37 °C with shaking (230 rpm). Bacterial growth was quantified by measuring the OD_600_ before and after 24 h of incubation, and cell viability at each dilution was confirmed by plating 10 μL aliquots onto nutritive agar plates, followed by static incubation at 37 °C overnight. Results were expressed as the percentage of growth inhibition. Furthermore, the formation of a protein corona on FAB NPs was evaluated by incubating 800 μL of NP suspension (5 mg/mL) with 200 μL of fetal bovine serum at 37 °C and 230 rpm for 10 min. The samples were centrifuged at 18,000 g for 10 min at 4 °C to pellet the NPs together with the adsorbed proteins, while unbound serum proteins remained in the supernatant. The protein-corona-coated NPs were resuspended in fresh medium and assessed for antibacterial activity as described above.^29^

### 2.5 Biofilm Inhibition

The ability of Fe_3_O_4_, FA, and FAB NPs to inhibit biofilm formation by *S. aureus*, *P. aeruginosa*, and *E. coli* was evaluated as described following a previously reported procedure.^19^ Briefly, overnight bacterial cultures in TSB were diluted to an OD_600_ of 0.01. Then, 50 μL of NPs at different concentrations (starting at 3 mg/mL) were mixed with 50 μL of bacterial suspension in 96-well microplates and incubated for 24 h under static conditions at 37 °C. After incubation, wells were washed with distilled water, and biofilms were fixed at 60 °C. Fixed biofilms were stained with 0.1% (w/v) crystal violet for 10 min, washed, and dried at 60 °C. The bound dye was solubilized with 200 μL of 30% (v/v) acetic acid, and the absorbance of 125 μL from each well was measured at 595 nm to quantify biofilm formation.

### 2.6 Quorum Sensing (QS) Inhibition

#### 2.6.1 QS Inhibition in Gram-Positive Bacteria

Two methicillin-resistant *S. aureus* strains were used: USA300agr IR P2-GFP, in which GFP is expressed under the *agr* P2 promoter (reporting QS signaling), and USA300agr IR P3-GFP, in which GFP is expressed under the *agr* P3 promoter (reporting activation of the RNAIII regulon). These reporter strains allow quantification of changes in agr signaling, which is normally triggered by autoinducing peptides and controls transcription of RNAIII, the main effector of agr-regulated virulence gene expression in *S. aureus*.^30^ Overnight cultures were grown in TSB supplemented with 4.5 μg/mL chloramphenicol (TSB-C) at 37 °C with shaking (230 rpm), diluted to OD_600_ ≈ 0.01 in TSB-C, and mixed with equal volumes (50 μL) of Fe_3_O_4_, FA, or FAB NPs (0.38 mg/mL) in 96-well microplates. After 24 h incubation at 37 °C with shaking, OD_600_ and GFP fluorescence (λ_ex_ = 485 nm, λ_em_ = 535 nm) were measured using a microplate reader (Infinite M200, TECAN, Austria). Controls included bacteria without NPs, NPs in TSB-C without bacteria, and media alone. GFP fluorescence was first normalized to OD_600_ to account for cell density, and then expressed relative to the fluorescence value measured for the untreated bacterial control.

#### 2.6.2 QS Inhibition in Gram-Negative Bacteria

*C. violaceum* (CECT 5999), a violacein-producing strain dependent on acyl-homoserine lactone (AHL), was used as reporter strain. In this strain, violacein production is regulated by an AHL-dependent LuxI/LuxR-type QS circuit (CviI/CviR), allowing assessment of interference with AHL-mediated signaling.^31^ Overnight cultures were grown in LB supplemented with kanamycin (25 μM), adjusted to OD_600_ ≈ 0.4, supplemented with AHL (333 μM) to induce QS, and mixed in a ratio 1:1 with Fe_3_O_4_, FA, or FAB NPs (0.38 mg/mL). After 2 h of incubation at 30 °C with shaking (230 rpm), cultures were diluted with fresh LB containing kanamycin and AHL (166 μM) to maintain QS activation and, and the resulting 3 mL cultures were incubated overnight under the same conditions. Negative and positive controls were prepared with or without NPs and AHL. Bacterial growth was assessed by measuring OD_600_, while bacterial viability was assessed by colony-forming unit (CFU) counting after preparing 10-fold serial dilutions in PBS (10^–1^ – 10^–8^) and plating on LB-kanamycin (25 μM) agar. For violacein quantification, each culture was centrifuged (20,000 g, 4 °C, 5 min); the resulting pellet was resuspended in 1 mL of ethanol, and the cells were lysed using ultrasound (Bandelin Sonopuls HD 2070) operating at 50% amplitude. After removal of cell debris by centrifugation, violacein content in the supernatant was quantified by measuring absorbance at 595 nm using an Infinite M200 microplate reader (TECAN, Austria). Violacein production was expressed as a percentage relative to the positive control (AHL-induced bacteria without NPs), following normalization to bacterial biomass.

### 2.7 SEM Analysis of Bacterial Morphology

The effect of FAB NPs on bacterial cell microstructure was examined by SEM. Overnight cultures of *S. aureus*, *P. aeruginosa*, and *E. coli* were grown in MHB at 37 °C, centrifuged, and washed twice with PBS. The cells were then resuspended in PBS to an OD_600_ of ∼ 0.5 and exposed to 0.38 mg/mL FAB NPs. The mixture was incubated for 4 h at 37 °C with shaking (230 rpm). After incubation, 3 mL of each sample were filtered through a sterile cellulose membrane (0.22 μm pore size). The retained bacterial cells were fixed using a solution of paraformaldehyde (2% v/v) and glutaraldehyde (2.5% v/v) in PBS. SEM images were acquired using a Zeiss Merlin field-emission scanning electron microscope (Jena, Germany) operated at 3 kV by the Microscopy and X-ray Diffraction Service (SMiDRX) of the Universitat Autonoma de Barcelona.

### 2.8 Membrane Interaction

The interaction of FAB NPs with bacterial membranes was investigated using Langmuir monolayers. A phospholipid mixture of PE and PG in an 8:2 molar ratio was used to mimic a Gram-negative bacterial membrane. Monolayers were formed in a KSV NIMA Langmuir–Blodgett trough (model KN2002) equipped with symmetric mobile barriers and a Wilhelmy plate for surface pressure measurement. The trough was mounted on an antivibration table and enclosed in an insulated chamber maintained at 21 ± 1 °C. Prior to each experiment, the trough and barriers were cleaned with chloroform and rinsed with Milli-Q water. Then, 30 μL of a 0.5 g/L phospholipid solution in chloroform was spread onto the PBS subphase in the absence or presence of FAB NPs. A control containing PBS and NPs without phospholipids was also included. After 15 min of solvent evaporation, the monolayers were compressed and π-A isotherms were recorded.

### 2.9 Cytotoxicity Against Mammalian Cells

The cytotoxicity of Fe_3_O_4_, FA, and FAB NPs was evaluated using human keratinocyte and fibroblasts cell lines. Following seeding, cells were exposed to the NPs (from 0.05 to 1.5 mg/mL) and incubated for 24 h. Cell viability was then assessed using the Alamar Blue assay, as previously described.^32^ Cell morphology and viability were further evaluated using the LIVE/DEAD Viability/Cytotoxicity Kit (Invitrogen, USA). Briefly, cells were incubated with calcein-AM and ethidium homodimer-1 according to the manufacturer’s instructions, washed with PBS to remove excess dye, and observed under a fluorescence microscope (NIKON Eclipse Ti–S, Japan).

### 2.10 Resistance Development Assay

The potential for resistance development of FAB NPs was evaluated against *S. aureus* and *E. coli* following standard procedures,^33^ using 2-fold serial dilutions in nutrient medium and visual inspection of bacterial growth after 24 h of incubation at 37 °C to determine the minimum inhibitory concentration (MIC). On Day 1, the MIC of FAB NPs was determined for both bacterial strains, whereas the MIC of ciprofloxacin was tested only for *S. aureus* and the MIC of ampicillin only for *E. coli*. From Day 2 onward, samples showing bacterial growth at the highest NPs or antibiotic concentration were used as the inoculum for the next day. The inoculum was diluted 1:50 in fresh MHB before performing the MIC assay for the subsequent day. This procedure was repeated for 55 consecutive days, and changes in the MIC relative to Day 1 were recorded daily to monitor the development of resistance.

### 2.11 Evaluation of FAB NPs in Packed Bed Columns for Water Disinfection

Prior to water filtration, FAB NPs were immobilized by magnetic retention using a magnetic layer comprising 61 neodymium magnets (3 mm diameter, N42 grade, chromium-plated magnetic spheres). The magnetic layer was loaded with 70 mg of FAB NPs by incubation in a NPs suspension prepared in ultrapure water, after which the coated layers were freeze-dried prior to use.

Water filtration experiments were carried out using 60 mL syringes without plungers as columns, packed with 12 g of activated carbon (AC; specific surface area 850 m^2^/g; MGR 800, Chiemivall, Spain), confined between two stainless-steel filters (120 mesh). Two magnetic layers were placed within the AC bed: a FAB NP-coated magnetic layer (∼6.6 g) positioned 1.5 g below the top of the bed and an uncoated magnetic layer (∼6.6 g) positioned 1.5 g above the bottom of the column (Figure S2). The bottom magnetic layer was included to capture and retain any NPs potentially released from the upper coated layer. In addition, a control filtration column containing only uncoated magnetic layers was used to confirm that bacterial removal was attributable to the presence of the NPs.

Filtration experiments were performed following a procedure using an adapted procedure based on previously reported methodology.^34^ A 100 mL of *E. coli* suspension (ATCC 25922; ∼ 10^7^ CFU/mL) was recirculated through the columns using a peristaltic pump at a flow rate of 8.6 mL/min. The bacterial suspension was continuously stirred at 230 rpm to prevent cell sedimentation. The efficiency of *E. coli* removal was evaluated by colony counting after 30 min, 1, 2, 4, and 18 h of filtration.

### 2.12 Statistical Analysis

All experiments were conducted in triplicate (n = 3) and results are presented as the mean values ± standard deviation (S.D.). Statistical analyses were performed using OriginPro 8.5 software (OriginLab, USA). One-way analysis of variance (ANOVA) followed by Tukey’s posthoc test was used to assess significant differences between groups, with a confidence level of 95% (*p* < 0.05).

## 3 Results and Discussion

### 3.1 Nanoparticles Characterization

Fe_3_O_4_, FA and FAB NPs were prepared through a stepwise synthesis. First, Fe_3_O_4_ NPs were obtained by coprecipitation using NaOH, followed by sequential Ag^0^ deposition from AgNO_3_, and subsequent baicalein functionalization. The initial ζ-potential of Fe_3_O_4_ NPs was – 29.6 ± 1.6 mV, which increased to +4.9 ± 1.3 mV upon the addition of AgNO_3_, indicating a significant surface modification due to Ag^+^ reduction and deposition leading to the formation of a heterostructured Fe_3_O_4_/Ag nanocomposite (FA).^35^ After the introduction of baicalein, the ζ-potential shifted to – 18.5 ± 1.9 mV, suggesting further surface functionalization and moderate colloidal stability of the resulting FAB NPs (Figure S3). TEM imaging revealed that all three types of NPs were predominantly spherical (Figure 1A, Figure S4A, Figure S5A) with average diameters ranging from 8 to 12 nm (Figure 1B, Figure S4B, Figure S5B). Aggregates were observed in all samples, likely due to magnetic interactions, though FAB NPs formed smaller aggregates (Figure 1A), possibly reflecting reduced magnetic interactions after Ag and baicalein incorporation. It is noteworthy that hydrodynamic size and polydispersity index are not reported here, as dynamic light scattering measurements are unreliable for strongly magnetic NPs.

**Figure 1:**
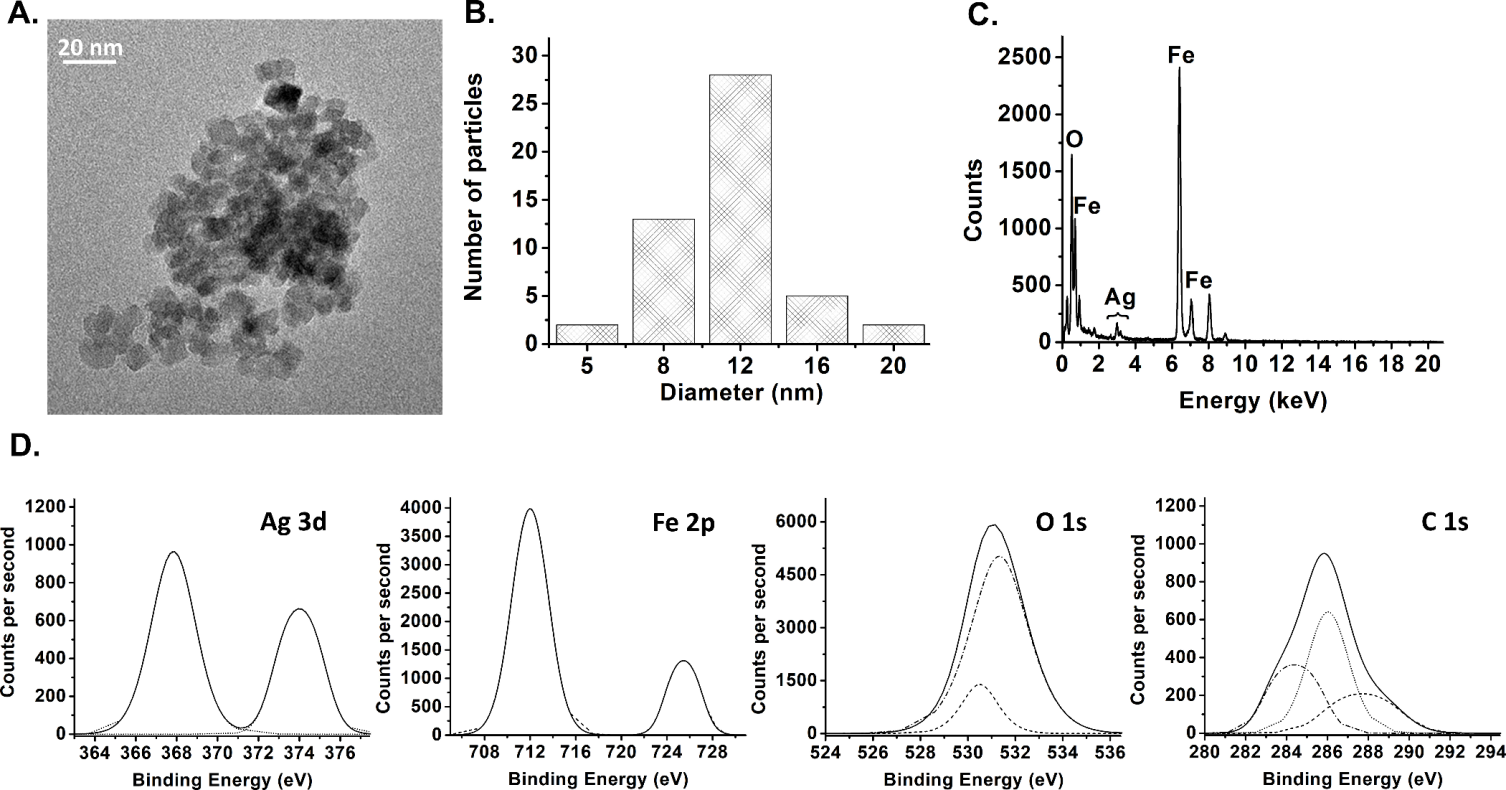
(A) TEM image, (B) size distribution data, (C) EDX spectrum, and (D) XPS spectra of FAB NPs.

EDX analysis demonstrated the presence of Fe, O, and Ag elements, confirming the incorporation of the metal on FAB (Figure 1C) and FA NPs (Figure S4C), while Fe_3_O_4_ NPs contained only Fe and O (Figure S5C). These results are consistent with the Ag 3d doublets at 367.2 – 367.8 eV (3d_5/2_) and 373.3 – 374.0 eV (3d_3/2_) in the XPS spectra, confirming the presence of metallic silver (Ag^0^) on the surface of both FAB (Figure 1D) and FA NPs (Figure S4D).^36^ In addition, the Fe 2p XPS spectrum of FAB (Figure 1D) exhibited peaks at ∼ 711–712 eV, reflecting an increased Fe^3+^/Fe^2+^ ratio, consistent with partial oxidation of Fe^2+^ associated with the reduction of Ag^+^ to Ag^0^.^37^ Similarly, FA NPs show similar Fe 2p features than FAB NPs (Figure S4D), while Fe_3_O_4_ NPs display the characteristic mixed Fe^2+^/Fe^3+^ contributions of magnetite (Figure S5D). XPS analysis of FAB NPs (Figure 1D) also revealed complex O 1s and C 1s envelopes, displaying a high-intensity O 1s peak around 531 eV (attributed to C–OOH groups), and a combination of nonoxygenated carbon (C–C at 284.4 eV) and oxygenated carbon species (C–O at 286 eV and C=O at 287.7 eV), confirming the presence of organic functionalities on the NPs surface derived from baicalein.^38^ In contrast, FA NPs showed a high-intensity O 1s peak at 530.2 eV, associated with Fe–O or Ag–O (Figure S4D), while Fe_3_O_4_ NPs displayed a high-intensity O 1s peak at 529.3 eV (Figure S4D) corresponding to the O-lattice of magnetite bonded to Fe^2+^/Fe^3+^.^39,40^

The successful incorporation of Ag^0^ and baicalein on FAB NPs, as confirmed by EDX and XPS, had a direct impact on their magnetic properties. As all three NPs (Fe_3_O_4_, FA, and FAB) are composed of magnetite, their magnetic behavior was first visually assessed using a neodymium magnet (for illustration purposes only), with all samples responding positively (Figure 2A). Quantitative evaluation was performed by VSM, with hysteresis loops showing sigmoidal magnetization curves, negligible coercivity (Hc ∼ 0) and remanence (*M*_r_ ∼ 0), characteristic for superparamagnetic behavior and full saturation at high fields. The normalized saturation magnetization (Ms) decreased in the order Fe_3_O_4_ NPs (37.6 ± 0.1 emu/g) > FA NPs (33.5 ± 0.1 emu/g) > FAB NPs (27.2 ± 0.1 emu/g). The drop in Ms for FA compared to Fe_3_O_4_ NPs is attributed to the presence of nonmagnetic Ag^0^, while the further decrease in FAB NPs is consistent with the addition of the organic, nonmagnetic baicalein (Figure 2B), with possible contributions from surface spin disorder and reduced crystallinity induced by Ag^0^ and baicalein.^41^

**Figure 2:**
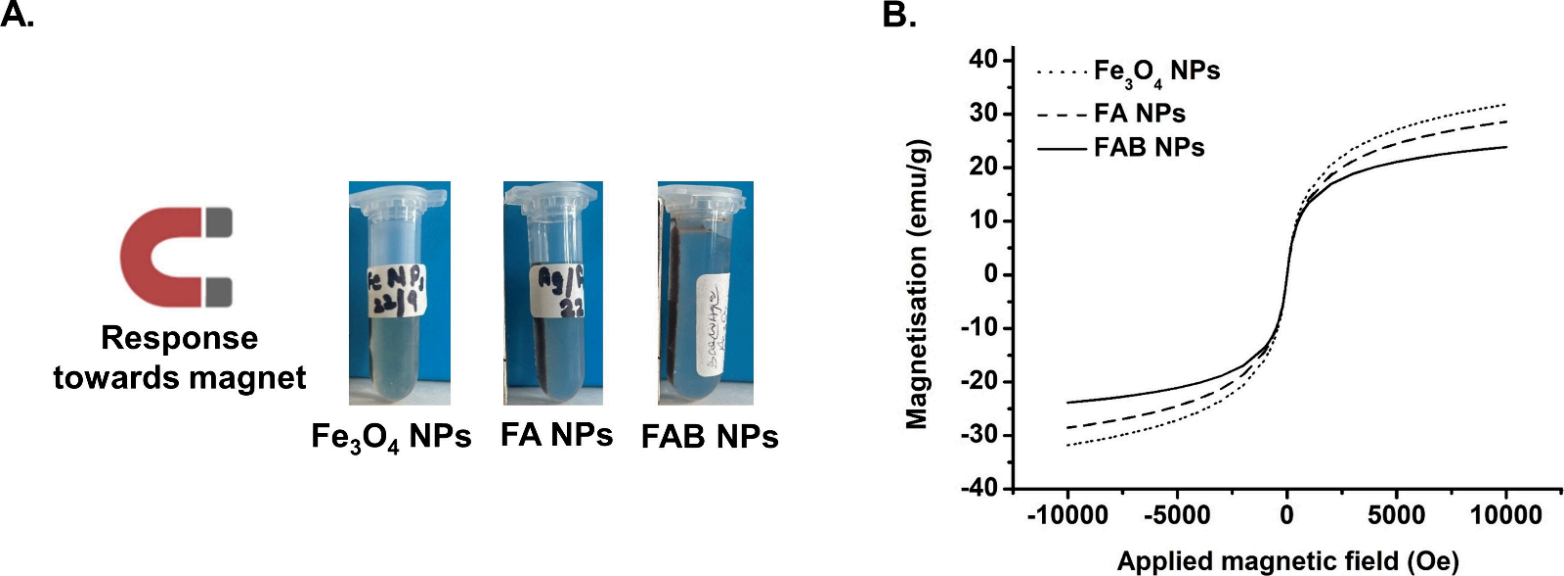
(A) Photographs of the NPs under a magnetic field. (B) VSM hysteresis loops showing the stepwise reduction in saturation magnetization for Fe_3_O_4_, FA and FAB NPs.

This decrease in Ms was further supported by structural characterization of the NPs. XRD spectrum of FAB NPs (Figure 3A) displays sharp peaks at 2θ values of 29.88°, 35.23°, 43.69°, 57.02°, 62.63°, corresponding to the (220), (311), (400), (511), and (440) planes, respectively. These peaks agree with the Fe_3_O_4_ NPs XRD pattern (Figure S6A) and are consistent with a face-centered cubic (FCC) structure. Additional peaks at 37.91° (111), 46.11° (200), 64.21° (220), and 77.12° (311) are present in FAB NPs, corresponding to FCC of Ag^0^, and are also observed in the XRD pattern of FA NPs (Figure S7A), confirming successful silver deposition. Notably, the peak at 53.14°, assigned to the (422) plane in Fe_3_O_4_ and FA NPs, is no longer evident in FAB NPs, potentially due to the presence of baicalein, which can reduce crystallinity,^42^ a factor known to contribute to decreased Ms in magnetic NPs.^41^ XRD parameters are summarized in Table S1. SAED analysis corroborated these findings, with FAB NPs exhibiting three diffraction rings corresponding to the 220, 311, and 111 planes (Figure 3B), whereas Fe_3_O_4_ NPs displayed six rings (220–511 planes) and FA NPs five rings in the same range (Figure S6B and Figure S7B). HRTEM imaging of FAB NPs revealed lattice fringes corresponding to 220, 311, 400, and 422 atomic planes (Figure 3C), confirming the crystal structure identified by XRD and SAED. Similar lattice fringe analysis was performed for Fe_3_O_4_ and FA NPs (Figure S6C and Figure S7C). Taken together, XRD, SAED, and HRTEM analyses indicate that the magnetite core structure remains practically intact, with minor loss of long-range crystallographic order. Therefore, these results support the conclusion that the observed decrease in Ms (Figure 2B) is not due to disruption of the crystal lattice, but rather to the incorporation of nonmagnetic Ag^0^ and baicalein layers, and potential surface spin disorder.

**Figure 3:**
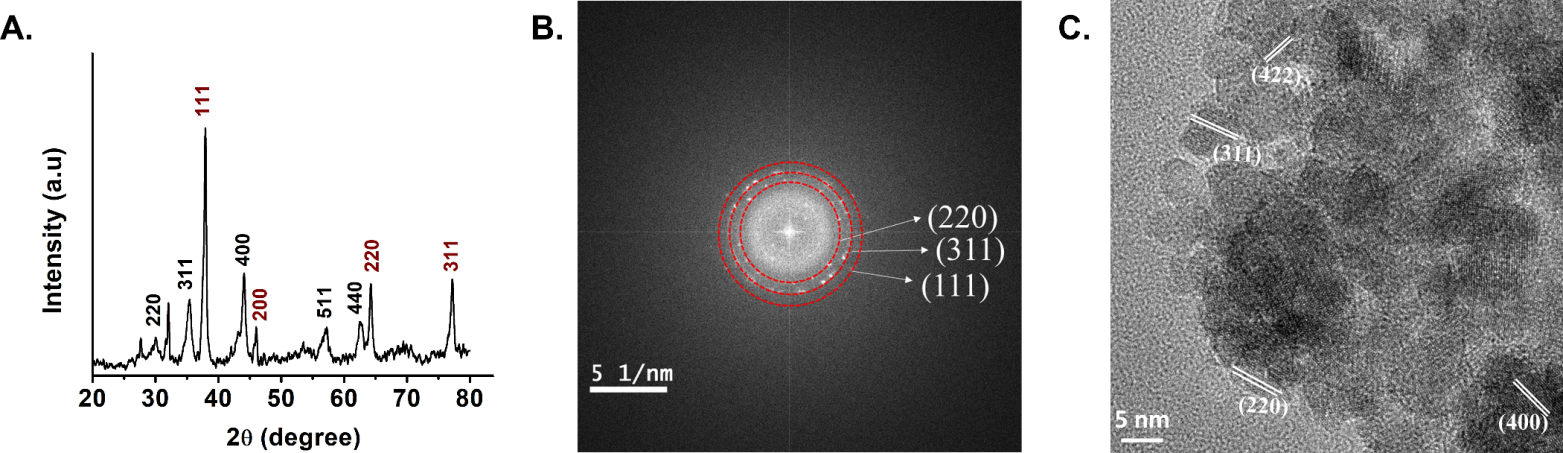
(A) XRD pattern, (B) SAED pattern, and (C) HRTEM images with lattice fringes of FAB NPs.

Fe_3_O_4_ and FA NPs showed no measurable phenolic content, indicating that neither the magnetite core nor the Ag^0^ coating contributes phenolic functionalities. In contrast, FAB NPs exhibited a significant increase in phenolic content from 0 to 0.77 μmol GAE/mg following baicalein functionalization, confirming the successful incorporation of the polyphenolic compound on the NPs surface (Figure 4A). The antioxidant activity of the three NPs across various concentrations revealed that Fe_3_O_4_ and FA NPs did not display radical-scavenging activity, indicating that neither the core nor the Ag^0^ shell contributes to DPPH inhibition, as expected. By contrast, FAB NPs exhibited dose-dependent antioxidant activity, with the highest inhibition observed at 1.5 mg/mL (∼43.8%) and decreasing to ∼ 0.83% at 0.05 mg/mL, highlighting the functional role of baicalein in imparting antioxidant properties (Figure 4B).

**Figure 4:**
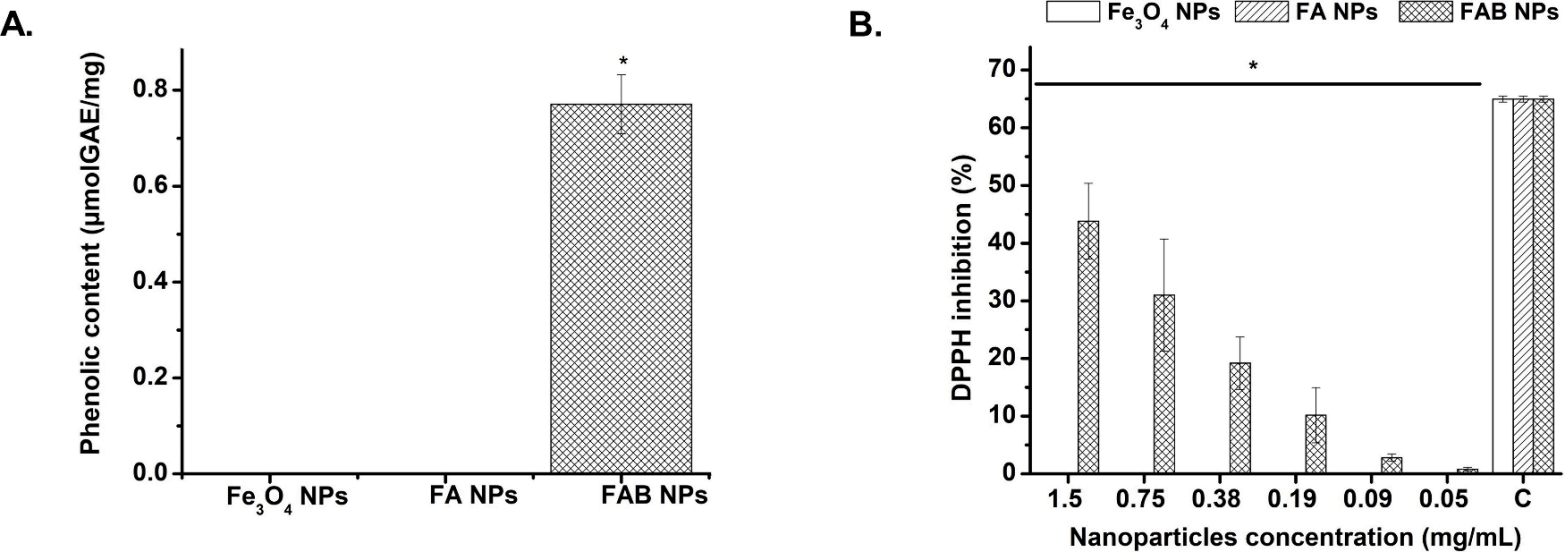
(A) Phenolic content and (B) antioxidant activity of Fe_3_O_4_, FA, and FAB NPs (C: ascorbic acid = 1 mg/mL).

### 3.2 Antibacterial Activity

The antimicrobial activity of the Fe_3_O_4_, FA and FAB NPs across various concentrations was tested against three relevant pathogens, with *E. coli* and *P. aeruginosa* representing ubiquitous environmental and opportunistic bacteria, and *S. aureus* providing additional relevance for biomedical and wastewater-associated contexts. Consistent with earlier reports, Fe_3_O_4_ NPs, demonstrated intrinsic antibacterial activity.^43^ However, the combination of Fe_3_O_4_ NPs with additional bioactive agents has been shown to further enhance their antimicrobial efficacy.^44,45^ In this study, FAB NPs exhibited significantly improved antibacterial profiles, achieving complete inhibition at 0.19 mg/mL for *S. aureus* and *P. aeruginosa*, and at 0.38 mg/mL for *E. coli*. These values were essentially identical to those obtained with FA NPs and lower than those of Fe_3_O_4_ NPs, which showed only partial inhibition even at 1.5 mg/mL (Figure 5). Therefore, baicalein functionalization did not reduce the antimicrobial performance of FA NPs, and both FA and FAB NPs can be considered broadly comparable in terms of antimicrobial efficacy. Importantly, FAB NPs preincubated in serum to allow protein-corona formation exhibited complete inhibition of all three evaluated strains at the same concentrations as untreated NPs, indicating that nonspecific protein adsorption does not compromise their antibacterial activity under protein-rich conditions. However, the flavonoid incorporation reduced the release rate of Ag^+^ and limited the cumulative release to 0.011 ± 0.002% (equivalent to 0.52 ± 0.11 mg/L) of the total silver content of FAB NPs after 7 days (168 h), indicating that baicalein effectively modulates and stabilizes silver availability (Figure 6). A plausible explanation, consistent with the known chemistry of flavonoids, is that the phenolic groups of baicalein interact with surface Ag^0^ through weak coordination or hydrogen-bonding interactions, and that its antioxidant character may reduce the extent of Ag^0^ oxidation. Both effects would contribute to a slower conversion of Ag^0^ into Ag^+^ and thus to the reduced release observed experimentally. Electrostatic or surface-stabilizing interactions between baicalein and the Ag domains may also play a role, although this cannot be conclusively established with the current data set. Importantly, the release profiles show a markedly lower cumulative Ag^+^ release in baicalein-functionalized samples, supporting the interpretation that baicalein modulates Ag^0^ surface reactivity rather than acting as a simple physical diffusion barrier. These results are consistent with previous reports describing surface functionalization with naturally occurring biomolecules as an effective strategy to improve the stability of metal-containing NPs.^9,21–24^ In contrast, FA NPs exhibited a sustained and progressive metal release, characterized by a gradual decline in release rate over time and the absence of a plateau within the experimental window, reaching a maximum cumulative Ag^+^ release of 0.362 ± 0.023% (equivalent to 1.53 ± 0.10 mg/L) of the total NPs silver content over the same period.

**Figure 5:**
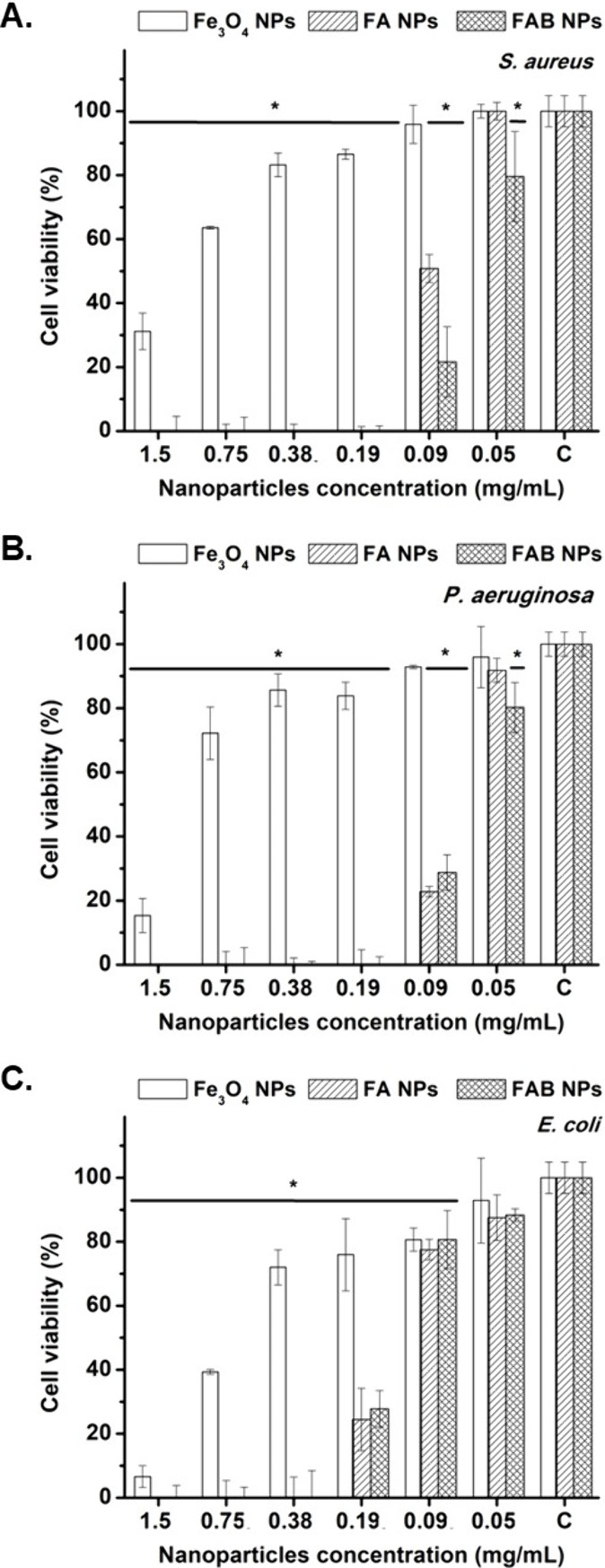
Bacterial cell viability (%) of *S. aureus* (A), *P. aeruginosa* (B), and *E. coli* (C) after exposure to different concentrations of Fe_3_O_4_, FA, and FAB NPs. “C” on the *x*-axis denotes the control group bacteria without NPs.

**Figure 6:**
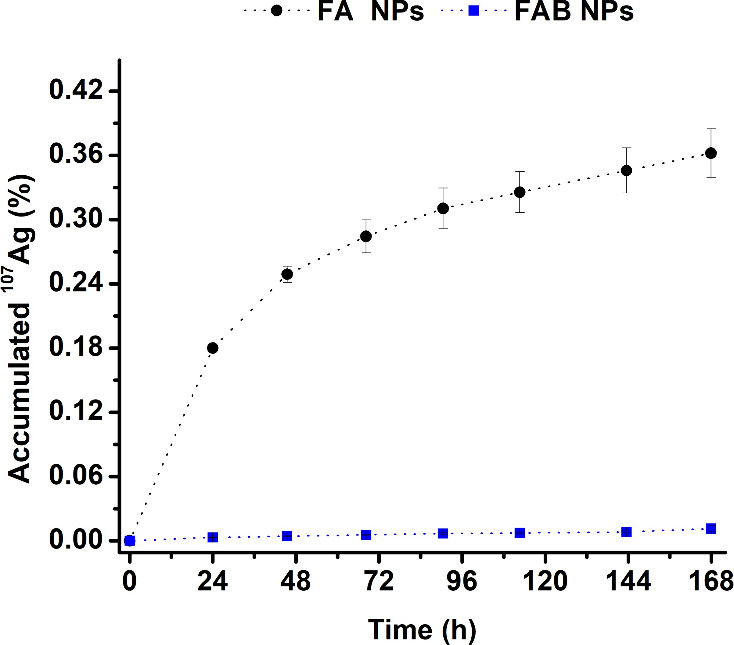
Cumulative Ag^+^ release profiles of FA and FAB NPs over 7 days (168 h).

Given that FAB and FA NPs display comparable antimicrobial activity across the three bacterial strains (Figure 5), despite the substantially lower Ag^+^ release of FAB NPs, these results suggest that baicalein contributes to the antibacterial effect, either directly or by facilitating Ag^+^ action, for example through membrane related effects. In line with this interpretation, baicalein has been reported to disrupt the inner and outer membranes of Gram-negative bacteria showing a synergistic effect with multiple antibiotics.^28^ More recently, the broad-spectrum antimicrobial activity of baicalein and its potential as an antibiotic adjuvant have been highlighted, further supporting the view that the flavonoid can enhance the antibacterial efficacy through complementary mechanisms.^46^ Overall, FAB NPs maintain the antimicrobial performance of FA NPs while reducing metal exposure, a combination that may translate into lower cytotoxicity and broader applicability in biomedical and environmental contexts.

### 3.3 Biofilm Inhibition Activity

Biofilms enhance pathogen persistence in clinical and environmental settings by increasing AMR, shielding cells from immune clearance, and facilitating horizontal gene transfer.^1–3^ FA and FAB NPs significantly inhibited biofilm establishment, whereas Fe_3_O_4_ NPs exhibited a weaker effect (Figure 7). Biofilm-forming capacity was significantly reduced (>60%) at concentrations of 0.75 mg/mL for *S. aureus* and 0.19 mg/mL for *P. aeruginosa* and *E. coli* following treatment with FA and FAB NPs. In contrast, Fe_3_O_4_ NPs required concentrations as high as 1.5 mg/mL to achieve biofilm inhibition of approximately 40 – 50% across all three tested pathogens. These results are consistent with those shown in Figure 5, as the antibacterial activity observed for all three NPs types is expected to have a corresponding impact on bacterial biofilm formation.

**Figure 7:**
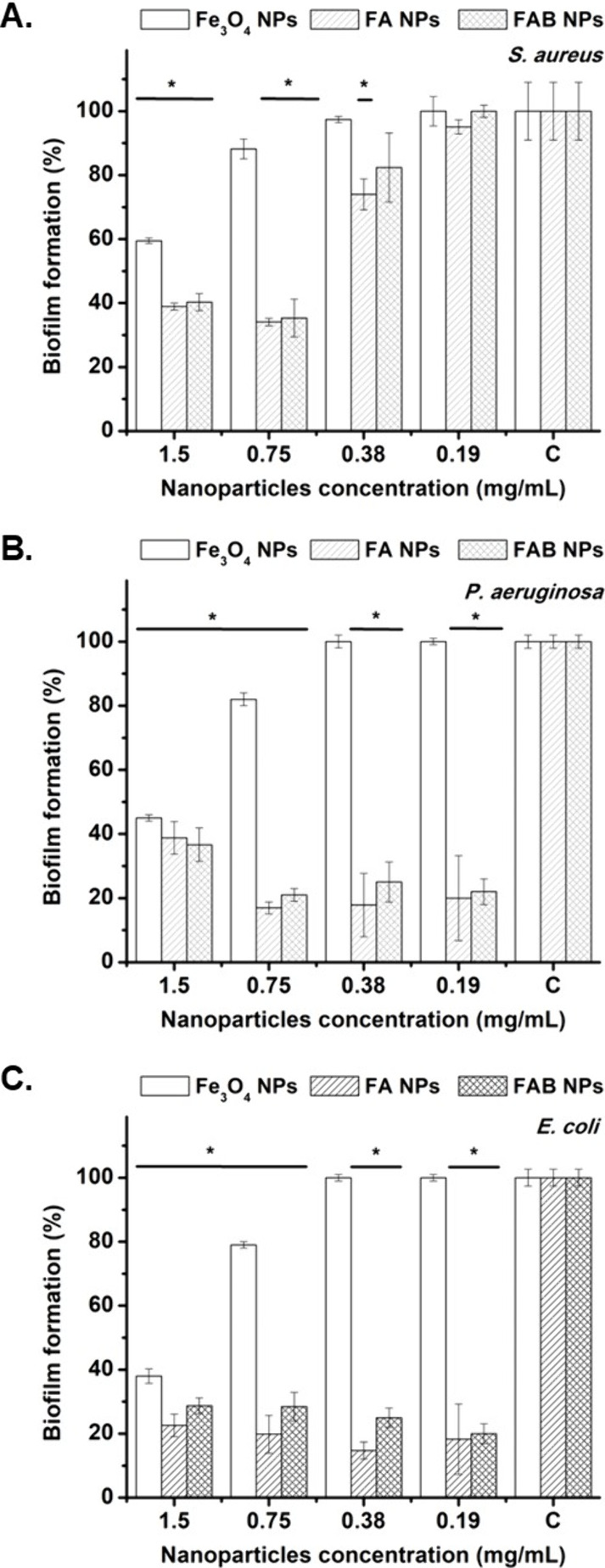
Biofilm formation of *S. aureus* (A), *P. aeruginosa* (B), and *E. coli* (C) after contacting them with FA, FAB, and Fe_3_O_4_ NPs. “C” on the *x*-axis denotes the control group.

Quorum sensing (QS) plays a fundamental role in the regulation of bacterial virulence and biofilm formation.^47^ In this context, the QS inhibition activity of Fe_3_O_4_, FA, and FAB NPs revealed clear distinctions between Gram-negative and Gram-positive systems (Figure 8). In *C. violaceum*, normalized violacein quantification confirmed that Fe_3_O_4_ NPs had no measurable effect on QS-regulated pigment production, whereas FA NPs suppressed violacein expression by approximately 25%. Under the same conditions, FAB NPs induced a more pronounced decline in violacein per viable cell, demonstrating a greater effect to QS disruption (Figure 8A). This effect could be attributed, at least in part, to the release of Ag^+^ ions from FA NPs (Figure 6). Silver-based NPs (e.g., polyvinylpyrrolidone- and citrate-coated Ag NPs) have been reported to disrupt QS by interfering with the biosynthesis and accumulation of AHL signal molecules, thereby attenuating violacein production in *C. violaceum*.^48–50^

**Figure 8:**
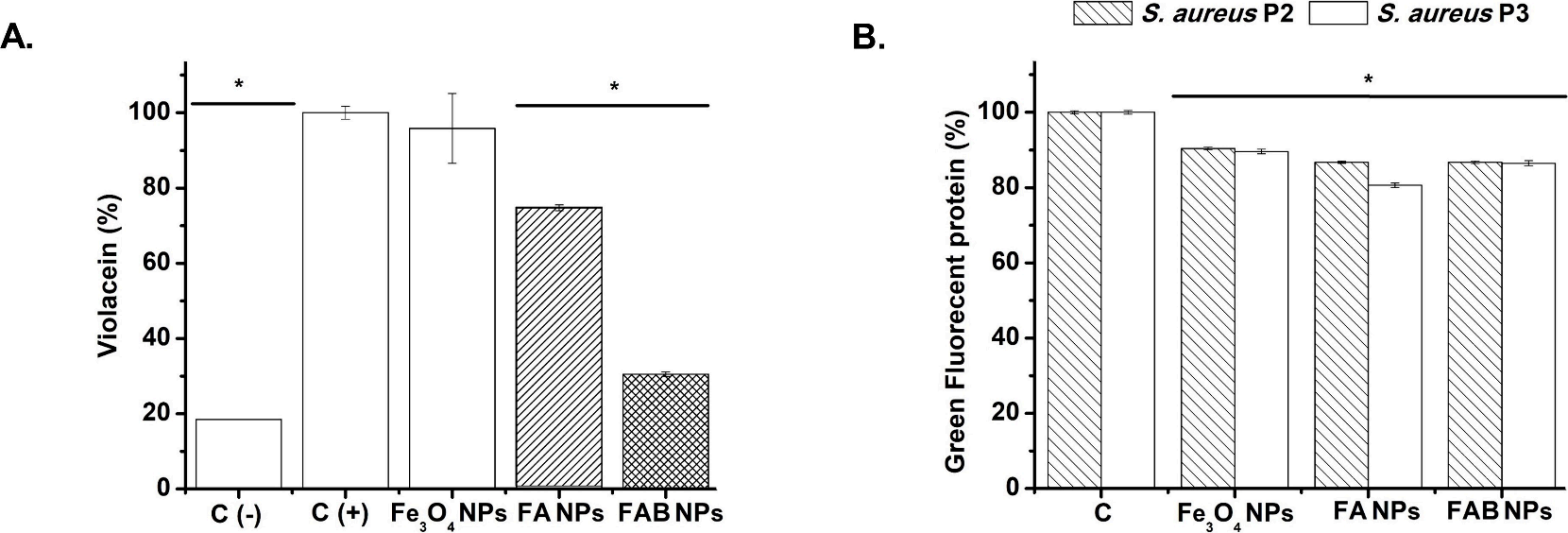
Inhibition of QS in (A) Gram-negative *C. violaceum* and (B) Gram-positive *S. aureus* (strains P2 and P3) following exposure to Fe_3_O_4_, FA, and FAB NPs. “C” on the *x*-axis indicates control groups.

In addition, baicalein has been described as a potent inhibitor of AHL-dependent QS pathways, acting on key molecular targets such as AHL synthases, receptor proteins, and downstream virulence regulators.^51–53^ In the case of FAB NPs, the simultaneous presence of silver and baicalein may result in additive or synergistic interference with QS signaling, contributing to the stronger suppression of violacein biosynthesis observed in *C. violaceum* (Figure 8A). A similar but more moderate trend was observed in both *S. aureus* QS reporter strains (P2 and P3), where FA and FAB NPs induced a statistically significant yet modest reduction in GFP expression compared to Fe_3_O_4_ NPs, suggesting that the presence of silver, and possibly partial Ag^+^ release, is required to induce QS inhibition in Gram-positive bacteria (Figure 8B). Notably, the comparable responses induced by FA and FAB NPs in both *agr* P2 and P3 reporters indicate that baicalein likely plays only a secondary role in the modulation of Gram-positive QS pathways, consistent with previous reports showing that silver-containing materials downregulate QS-associated genes in staphylococci.^54^ The attenuated QSI response observed in *S. aureus*, relative to Gram-negative models, may reflect fundamental differences in cell envelope architecture, as Gram-positive bacteria possess a substantially thicker peptidoglycan layer enriched with teichoic acids that can limit NPs interaction and Ag^+^ penetration, thereby reducing the impact on intracellular signaling processes such as QS regulation.^55^ Therefore, the differences observed in biofilm inhibition among Fe_3_O_4_, FA and FAB NPs treatments (Figure 7) result from a combined disruption of QS signaling (Figure 8) and alterations in bacterial population density (Figure 5).

To further investigate the mechanisms underlying biofilm and antimicrobial activity, SEM analyses were conducted on bacteria after exposure to FAB NPs, as this novel formulation displayed significant antimicrobial and antibiofilm activity, along with marked inhibition of QS, and lower Ag^+^ release. SEM observations revealed significant bacterial morphological alterations and NP-cell interactions, providing visual evidence that supports the observed microbial and biofilm suppression (Figure 9).

**Figure 9:**
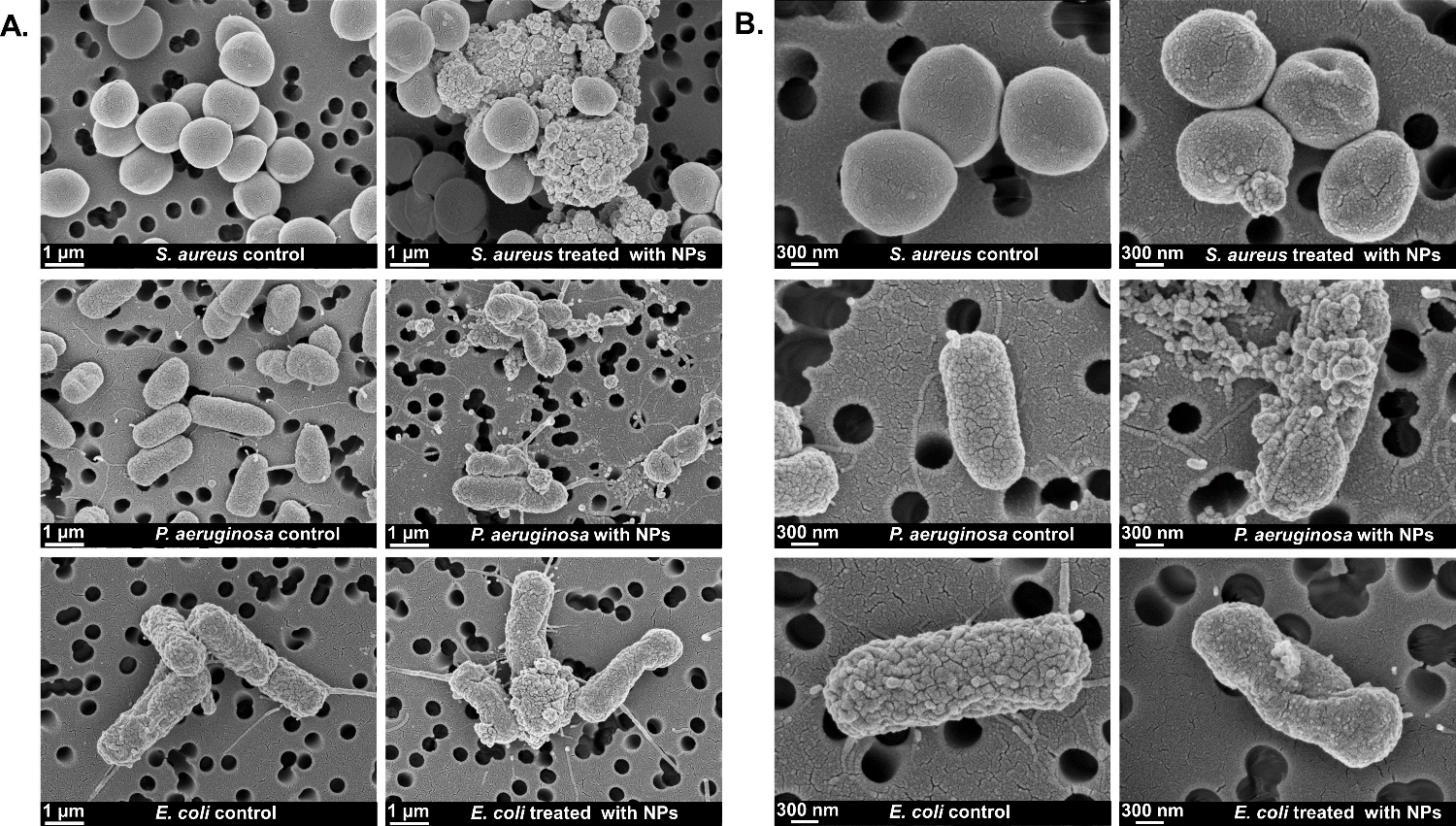
SEM image of *S. aureus*, *P. aeruginosa*, and *E. coli* without NPs treatment (control) and treated with FAB NPs at 30,000x (A) and 70,000x (B) magnifications.

Untreated *S. aureus*, *P. aeruginosa*, and *E. coli* displayed their characteristic morphologies, coccal for staphylococci and rod-shaped for bacilli, with smooth surfaces consistent with intact and healthy cells. Following incubation with FAB NPs, all three species exhibited extensive ultrastructural damage, including membrane collapse, surface roughening, pronounced deformation, and reduced cell volume. Gram-negative *E. coli* and *P. aeruginosa* additionally showed bleb formation, appearing as small vesicle-like protrusions from the outer membrane, a hallmark of severe cellular stress and a well-recognized defense response to toxic insults and oxidative damage.^19,56^ Similarly, *S. aureus* cells displayed marked deformation and structural disruption. These alterations reflect severe cellular stress and are likely to impair bacterial attachment, providing a mechanistic explanation for both the observed antimicrobial activity and biofilm inhibition.

To further probe the underlying mechanism of FAB NPs-membrane interactions suggested by the SEM observations, Langmuir monolayer analyses were performed (Figure S8). FAB NPs alone exhibited a flat isotherm, indicating no inherent surface activity or monolayer formation. However, when inserted into a PEPG monolayer, a lateral expansion of approximately 12 cm^2^ was observed without significantly altering the collapse pressure relative to pristine PEPG. This right-shift in the π-A isotherm indicates that FAB NPs intercalate into the phospholipid monolayer, disrupting molecular packing while maintaining overall film stability. Such behavior parallels previous reports showing that hybrid or functionalized nanomaterials can intercalate into phospholipid assemblies, disturbing lipid–lipid interactions.^20^ The baicalein moieties of FAB NPs, rich in aromatic functionalities, are likely to enhance NP-lipid affinity through hydrophobic insertion and π-π or hydrogen-bonding interactions, thereby promoting monolayer expansion. This displacement effect, observed at biologically relevant lateral pressures (i.e., 30 – 35 mN/m), aligns with a membrane-perturbing mechanism and supports the notion that FAB NPs may exert part of their antimicrobial action through modulation of lipid packing and increased membrane disorder. Collectively, these findings corroborate the SEM data, demonstrating that FAB NP-induced alterations in membrane integrity can interfere with the early attachment phase of biofilm formation.^57–60^ The convergence of antimicrobial activity, FAB-mediated QS suppression, and membrane disruption provides a coherent mechanistic basis for the observed reduction in biofilm formation across bacterial species. By impairing cell morphology and compromising membrane integrity, FAB NPs consequently interfere with early attachment, hindering the transition from planktonic to sessile lifestyles. This combined effect, effectively suppressing biofilm development and bacterial aggregation on surfaces, may contribute to the mitigation of AMR.^7,61,62^

### 3.4 Cytotoxicity Assessment in Mammalian Cells

As demonstrated in previous sections, both FA and FAB NPs exhibit pronounced antimicrobial and biofilm inhibition activity, highlighting their potential applications in bacterial disinfection and infection control.^63^ For silver-based nanomaterials to be considered for biomedical use, it is essential to balance antimicrobial efficacy with adequate biocompatibility toward mammalian cells. Therefore, the cytotoxicity of Fe_3_O_4_, FA, and FAB NPs was evaluated after 24 h of incubation using human fibroblasts and keratinocytes (Figure 10). Fe_3_O_4_ NPs showed consistent cell viability across all the concentrations tested proving high biocompatibility. This observation agrees with their well-documented use in biomedical applications, including drug delivery, magnetic resonance imaging, and hyperthermia, where biocompatibility is a critical requirement.^64^ By contrast, FA NPs displayed high cytotoxicity in both model cells even at 0.05 mg/mL. Notably, FAB NPs maintained more than 80% cell viability up to 0.75 mg/mL, indicating that baicalein capping effectively mitigates cytotoxic effects. This improved biocompatibility can be attributed to the substantially reduced Ag^+^ release observed for FAB NPs (Figure 6), as lower metal ion exposure is generally associated with decreased cytotoxicity in mammalian systems.^65^ In addition to limiting Ag^+^ release, FAB NPs were shown to exhibit intrinsic antioxidant properties (Figure 4), which further contribute to their favorable profile. This multifunctional behavior is particularly relevant, as oxidative stress is a key contributor to the cytotoxicity commonly associated with silver-based nanomaterials.^63^ The antioxidant capacity of FAB NPs may therefore play a complementary role in mitigating Ag-induced cellular damage, acting alongside the reduced Ag^+^ release to preserve mammalian cell viability.

**Figure 10:**
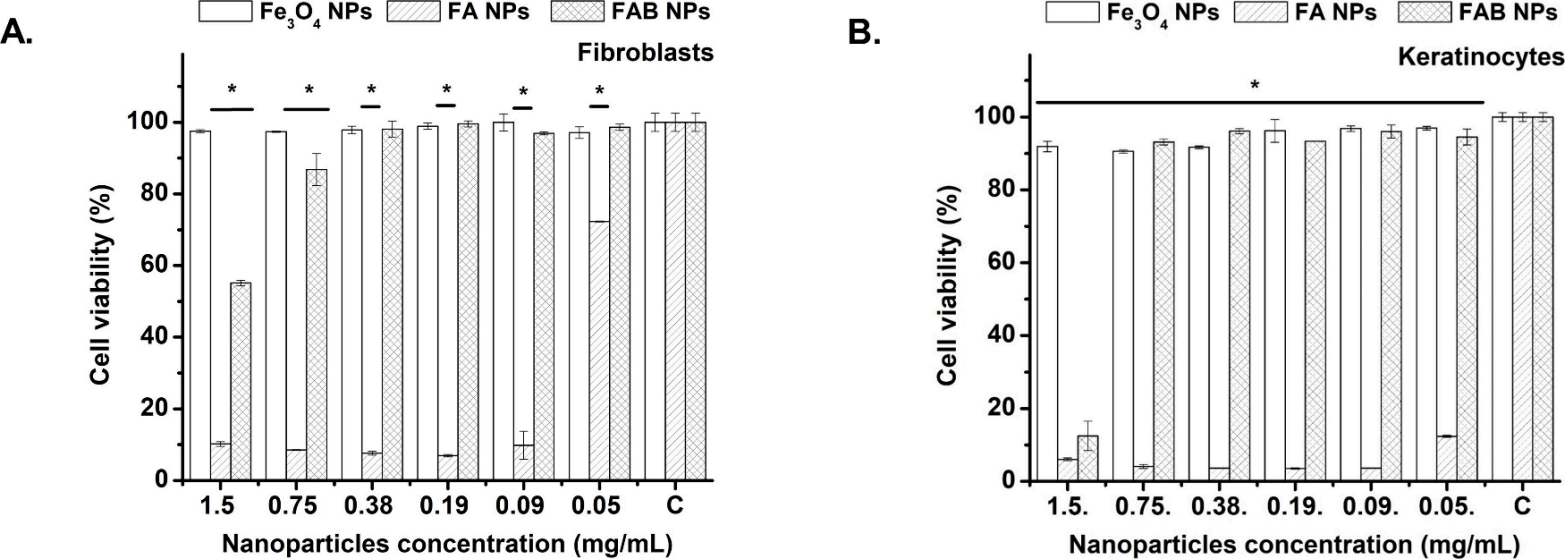
*In vitro* cytotoxicity of Fe_3_O_4_, FA, and FAB NPs at various concentrations following 24 h incubation with human fibroblasts (A) and keratinocytes (B). “C” on the *x*-axis denotes the control group.

Consistently, the reduced toxicity of FAB NPs was further supported by the absence of detectable alterations in cell morphology, which was comparable to that observed for silver-free Fe_3_O_4_ NPs (Figure 11). By effectively decoupling antibacterial efficacy from cytotoxic effects, baicalein functionalization emerges as a promising strategy to engineer safer silver-based nanomaterials. Overall, the combination of antimicrobial, antioxidant, biocompatible, and magnetic properties position FAB NPs as attractive candidates for biomedical applications, including magnetic-assisted antimicrobial coatings, wound-related materials, and other therapeutics where bacterial control and host cell viability are crucial.

**Figure 11:**
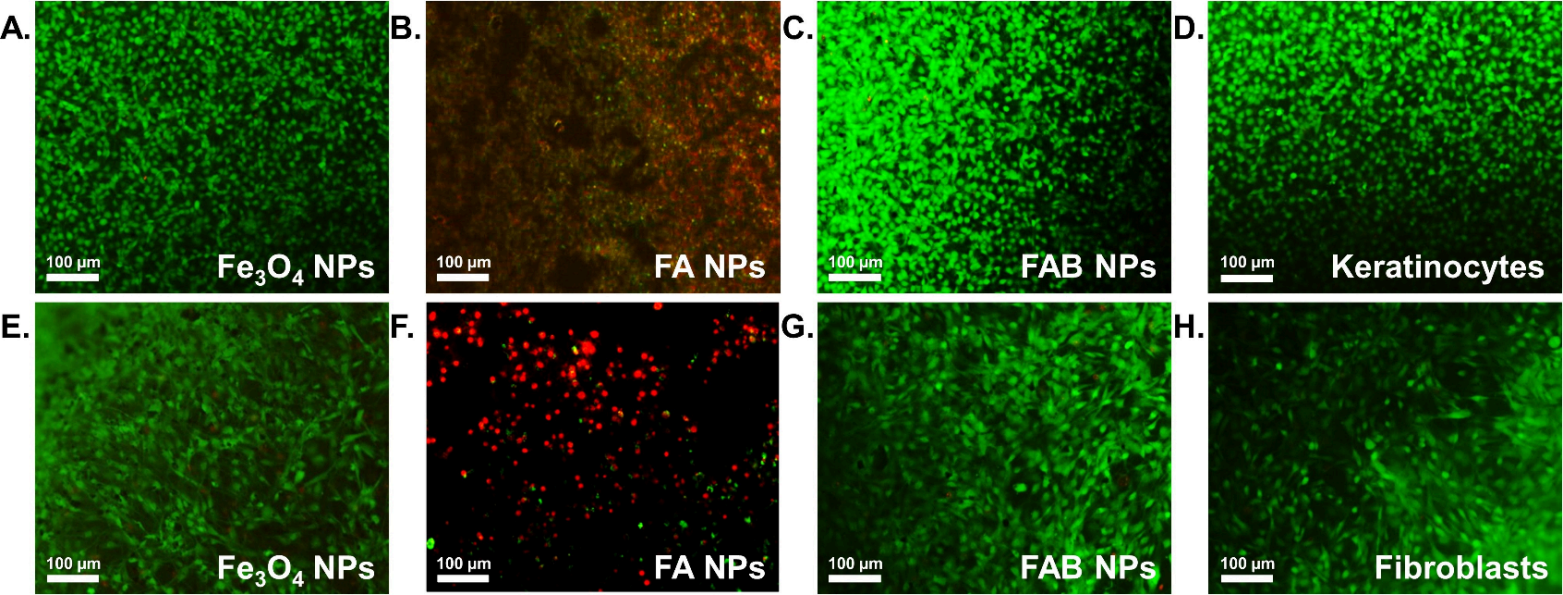
Representative fluorescence microscopy images of human keratinocytes (A-D) and fibroblasts (E-H) following 24 h incubation with Fe_3_O_4_, FA, FAB NPs, or without NPs (controls; D and H). Cells were stained using calcein-AM and ethidium homodimer-1 to visualize live (green) and dead (red) cells, respectively.

### 3.5 Resistance Development

Given the multiple antibacterial mechanisms exhibited by FAB NPs, we hypothesized that they may prevent the emergence of bacterial resistance. AMR is a naturally occurring process in which bacteria develop the ability to withstand the effects of a specific antimicrobial agent after prolonged exposure. Conventional antibiotics typically target specific cellular processes or molecules essential for bacterial survival. When exposed to an antibiotic, susceptible bacteria are inhibited or eliminated, while those harboring resistance traits persist.^17,18^ The World Health Organization (WHO) has identified AMR as a major global health threat requiring immediate attention.^66^

To evaluate resistance development, *S. aureus* and *E. coli* were selected as representative Gram-positive and Gram-negative model organisms, respectively. Both strains were continuously exposed for 55 days to either FAB NPs or conventional antibiotics to which they are known to be susceptible, i.e., ciprofloxacin for *S. aureus* and ampicillin for *E. coli*. Following prolonged antibiotic exposure, the MICs increased by 4096- and 256-fold for ciprofloxacin (*S. aureus*) and ampicillin (*E. coli*), respectively (Table 1), indicating the acquisition of resistance. In contrast, exposure to FAB NPs resulted in only a 2-fold increase in MIC for both bacterial strains, which is minimal and does not indicate significant resistance development.^67^

**Table 1:** Alterations in MIC for *S. aureus* and *E. coli* after 55 Days of Antibiotic or FAB NPs Exposure

	MIC Value Change^a^	
Material	*S*. *aureus*	*E. coli*
**FAB NPs**	2	2
**Ciprofloxacin**	4096	*-*
**Ampicillin**	-	256

a Values represent fold changes in MIC relative to Day 1 (e.g., a value of 2 indicates a 2-fold increase in MIC).

Previous studies have explored alternative antibacterial agents, including silver-based and metal oxide NPs.^8,68^ However, some bacterial strains have been reported to acquire resilience against Ag^+^ and even Ag NPs. Designing effective antibacterial materials that minimize the emergence of resistant strains remains a major challenge.^69,70^ FAB NPs, however, exhibit multiple and nonspecific mechanisms of action, such as direct interactions with bacterial surfaces (Figure S7), which likely reduce the probability of resistance development compared to conventional antibiotics. In combination with their demonstrated biocompatibility toward keratinocytes and fibroblasts (Figures 10 and 11), these properties position FAB NPs as attractive candidates for the design of novel therapeutic agents, including topical formulations for skin-related infections and antimicrobial coatings for catheters or implantable devices.

### 3.6 Water Disinfection Efficiency of FAB NPs in Packed Bed Columns

Having demonstrated the multifunctional properties of FAB NPs, including strong antimicrobial and antibiofilm activities, we next explored their potential for environmental applications. Water disinfection remains a significant challenge due to the formation of viable biofilms on conventional filters, such as AC, which can limit disinfection efficiency and potentially contribute to the development of AMR.^71^ Therefore, FAB NPs were incorporated into lab-scale packed-bed columns using a neodymium magnetic layer coated with the NPs. Disinfection performance was evaluated using *E. coli* as the target microorganism, given its widespread use as a reference indicator of faecal contamination and its relevance as a major waterborne pathogen of public health concern.^72^ As shown in Figure 12, complete elimination of *E. coli* from artificially contaminated water was achieved after 8 h of sample recirculation. As expected, AC control columns demonstrated poor performance in *E. coli* removal, achieving less than a 1-log reduction in bacterial count in the filtered samples after 18 h of recirculation. This limited removal was mainly attributed to bacterial adsorption onto the filter media rather than to an active disinfection mechanism. Additionally, the use of FAB NPs as a filler in AC columns for water disinfection proved to be environmentally safe, as the concentration of Ag^+^ detected in the treated effluent remained below the detection limit of the ICP-MS method (LOD < 0.25 μg/L). This finding mitigates concerns regarding the potential environmental impact associated with the release of toxic silver species from FAB NPs into treated water. These results are consistent with the enhanced stability observed for baicalein-functionalized NPs, as shown in Figure 6. Furthermore, the WHO has established a guideline value for Ag^+^ in drinking water of 100 μg/L, based on potential aesthetic effects on water physical properties, as no significant health risks have been identified at Ag^+^ concentrations below this threshold.^72^

**Figure 12:**
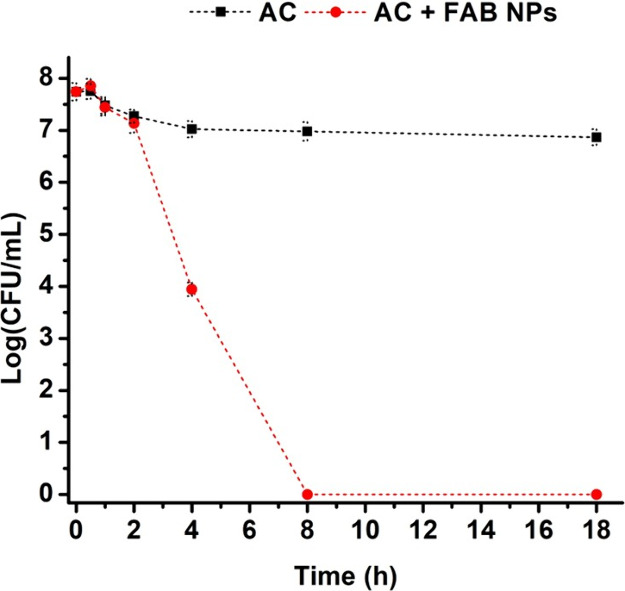
*E. coli* removal from contaminated water in lab-scale packed-bed columns filled with activated carbon (AC) or AC mixed with FAB NPs during 18 h of recirculation.

It is also worth noting that the bacterial load used to feed the columns in this study (∼10^7^ CFU/mL) was significantly higher than the microbial concentrations typically detected in real secondary effluents, which generally range from 10^3^ to 10^6^ CFU per 100 mL.^34^ Therefore, under more realistic operational conditions, it is expected that a filtration system incorporating FAB NPs would exhibit even higher disinfection efficiency, while reducing treatment time and probably extending column service life. Overall, this work demonstrates that advances in the engineering of nanocomposites integrated into water filtration systems can promote sustainable and effective water treatment solutions with strong potential for real-life applications.

## 4 Conclusions

In this study, we developed a baicalein-functionalized Fe_3_O_4_/Ag nanocomposite (FAB NPs) that integrates antimicrobial efficacy, magnetic responsiveness, antioxidant activity, and enhanced biocompatibility into a single multifunctional platform. The baicalein coating played a key role in stabilizing the nanocomposite, significantly reducing Ag^+^ release while preserving strong antibacterial, antibiofilm, and quorum sensing (QS) inhibition activities against both Gram-positive and Gram-negative pathogens. Mechanistic investigations demonstrated that FAB NPs interact directly with bacterial membranes, induce severe morphological damage, and disrupt QS pathways, thereby impairing bacterial viability and biofilm formation through multiple, nonspecific mechanisms. This multimodal mode of action translated into a negligible tendency to induce bacterial resistance during prolonged exposure, in contrast to conventional antibiotics. Importantly, baicalein functionalization improved cytocompatibility toward human keratinocytes and fibroblasts, likely due to the combined effects of reduced Ag^+^ ion release and intrinsic antioxidant activity. Beyond biomedical relevance, the successful integration of FAB NPs into packed-bed filtration columns enabled efficient water disinfection with minimal Ag^+^ leaching, highlighting their potential for safe and sustainable environmental applications. Overall, this work underscores the potential of biofunctionalized magnetic silver-based nanocomposites as versatile, resistance-mitigating antimicrobial materials, opening new avenues for their implementation in infection control, biomedical devices, and advanced water treatment technologies.

## Supplementary Material



## Data Availability

The authors confirm that all data supporting the findings of this study are provided within the article.

## Notes

Diffraction parameters, surface pressure–area isotherms, XRD and SAED patterns, HRTEM images, size distribution data, EDX spectrum, XPS spectra, zeta potential, schematic illustrations of activated carbon packed column and synthetic scheme (PDF)
