# Supplementary material for: Harnessing the Antimicrobial Potential of Baicalein-Coated Fe3O4/Ag Nanoparticles for Biomedical and Environmental Applications
Source: ACS Appl Mater Interfaces. 2026 Apr 24;18(17):25491–504. doi: 10.1021/acsami.6c02389 (PMC13154117; doi:10.1021/acsami.6c02389)
Supplement: Supplementary file 1 [file am6c02389_si_001.pdf]

Supplementary Information

Harnessing the Antimicrobial Potential of Baicalein-Coated Fe_3O_4/Ag Nanoparticles for Biomedical and Environmental Application

Jeniffer Blair[1], Garima Rathee[1], Antonio Puertas-Segura[1], Kristina Ivanova[1], Aleksandra Ivanova[2], Leonardo Martín Pérez[1], Tzanko Tzanov[1*]

[1]Grup de Biotecnologia Molecular i Industrial, Departament d'Enginyeria Química, Universitat Politècnica de Catalunya, Rambla de Sant Nebridi 22, 08222 Terrassa (Barcelona), Spain.

[2]Cleantech Bulgaria Foundation. 11 Lukashov str., floor 6, Vratsa 3000, Bulgaria

*Author to whom correspondence should be addressed.

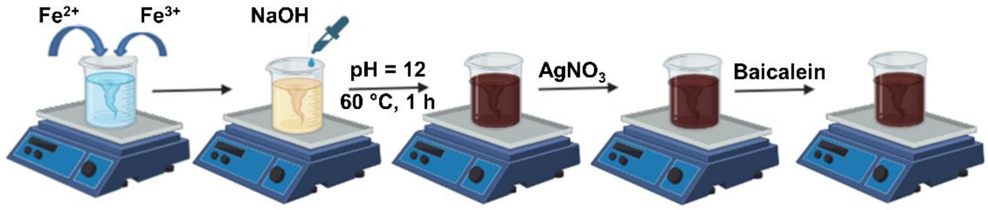

Fig. S1. Schematic illustration of the synthesis of Fe_3O_4, FA, and FAB NPs.

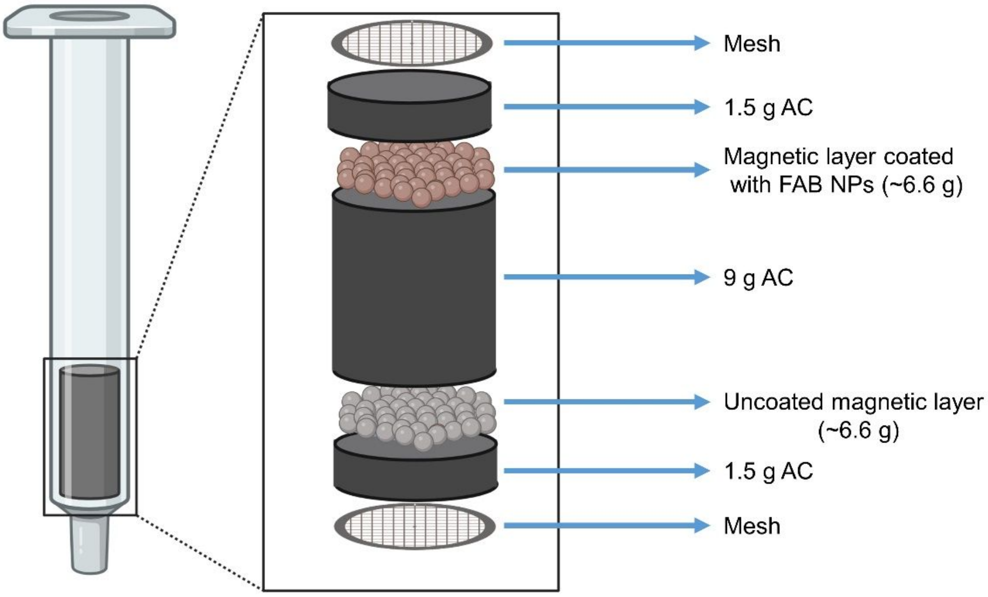

Fig. S2. Schematic illustration of the activated carbon (AC) packed column with two neodymium magnetic meshes (one coated with FAB NPs and another uncoated) fixed between two stainless steel meshes (120 Mesh).

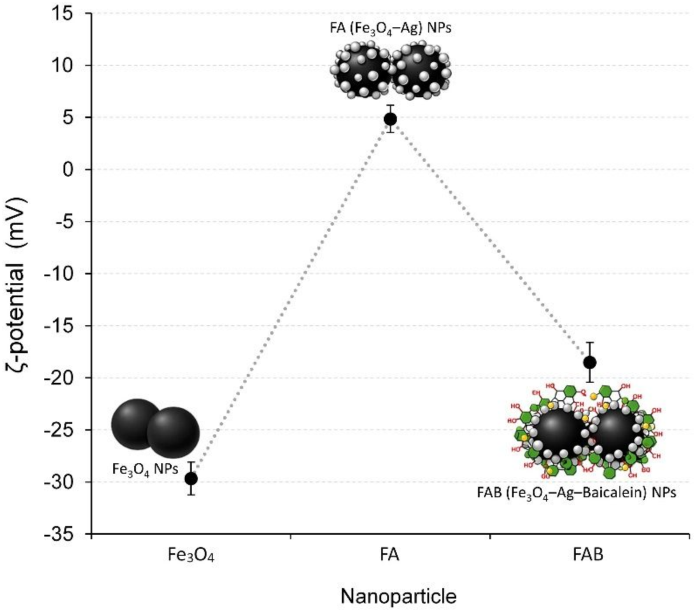

Fig. S3. ζ-potential of Fe_3O_4, FA, and FAB NPs.

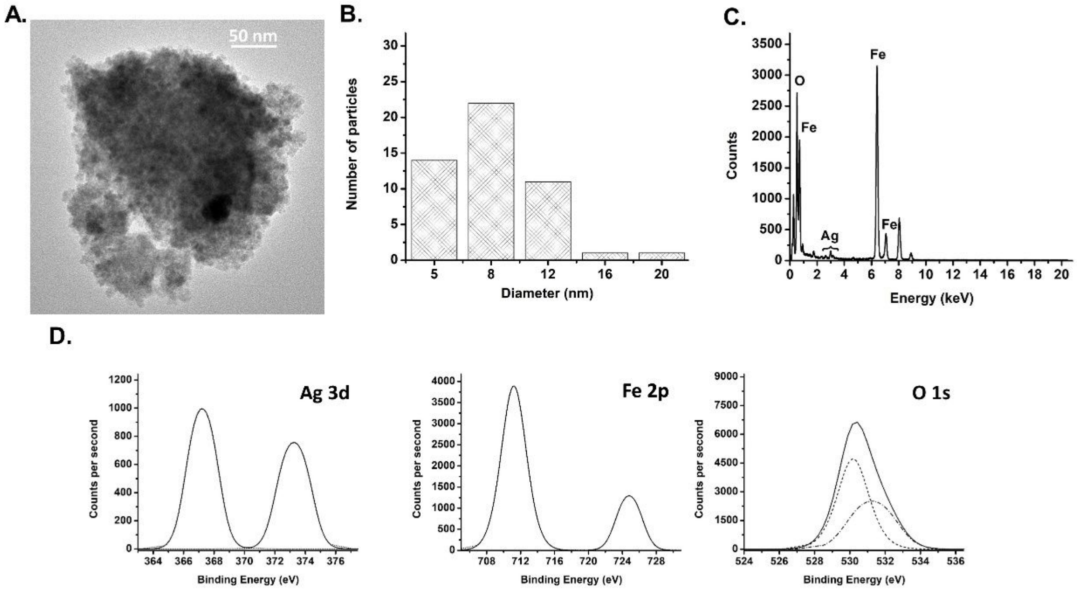

Fig. S4. (A) TEM image, (B) size distribution data, (C) EDX spectrum, and (D) XPS spectra of FA NPs.

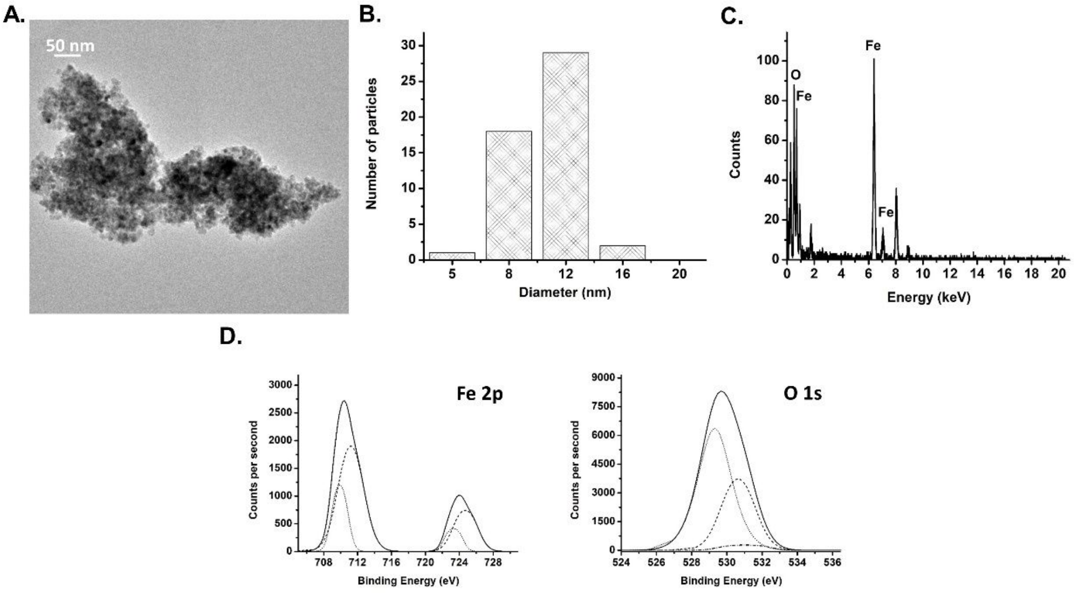

Fig. S5. (A) TEM image, (B) size distribution data, (C) EDX spectrum, and (D) XPS spectra of Fe_3O_4 NPs.

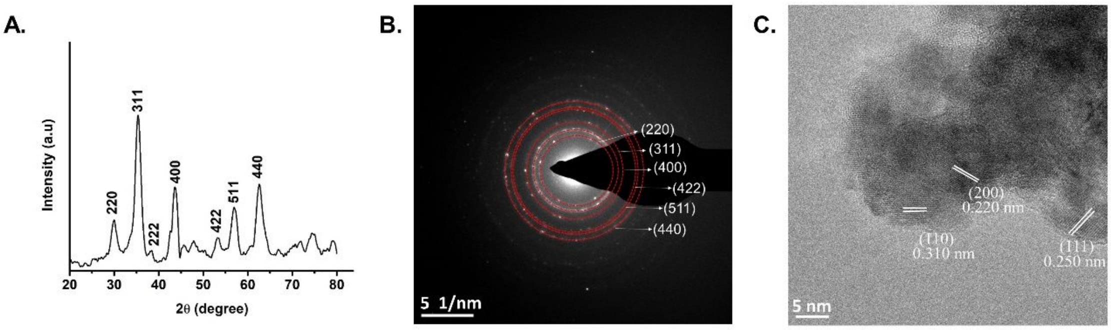

Fig. S6. (A) XRD pattern, (B) SAED pattern, and (C) HRTEM images with lattice fringes of Fe_3O_4 NPs.

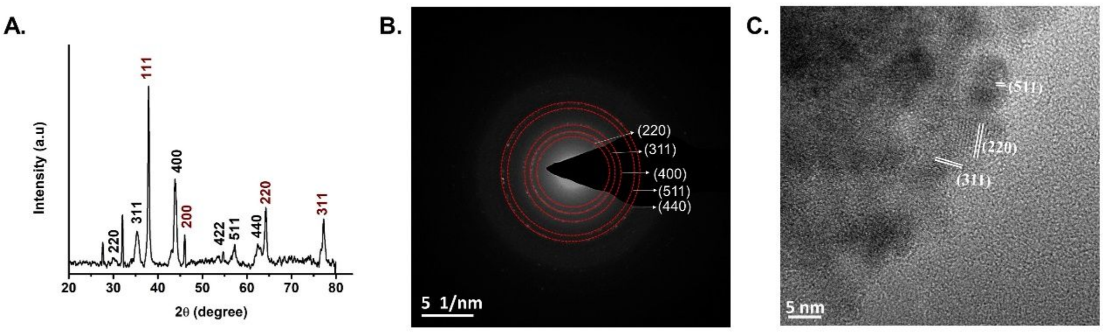

Fig. S7. (A) XRD pattern, (B) SAED pattern, and (C) HRTEM images with lattice fringes of FA NPs.

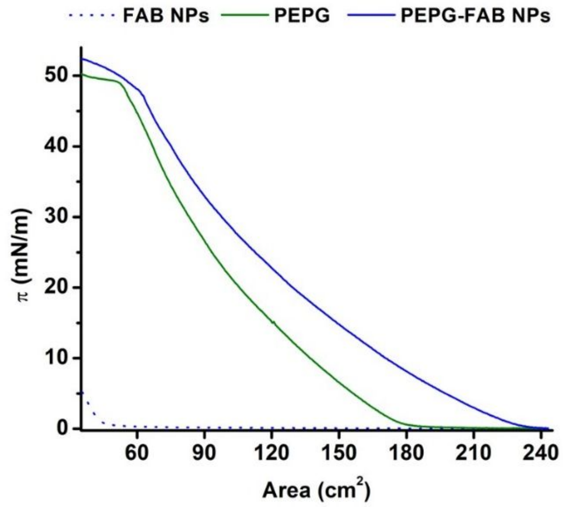

Fig. S8. Surface pressure-area isotherms of phosphatidylethanolamine and phosphatidylglycerol mixture monolayer (PEPG), FAB NPs, and PEPG with FAB NPs (PEPG-FAB NPs).

Table S1. Diffraction parameters of Fe_3O_4, FA and FAB NPs.

**2θ**	**Miller indices**			**d-spacing/nm**
	**h**	**k**	**l**	
**Fe_3O_4 NPs**				
29.88	2	2	0	0.298
35.23	3	1	1	0.255
38.33	2	2	2	0.234
43.69	4	0	0	0.207
53.14	4	2	2	0.172
57.02	5	1	1	0.161
62.63	4	4	0	0.149
**FA NPs**				
29.88	2	2	0	0.298
35.23	3	1	1	0.255
37.91	1	1	1	0.237
43.69	4	0	0	0.207
46.11	2	0	0	0.197
53.14	4	2	2	0.172
57.02	5	1	1	0.161
62.63	4	4	0	0.149
64.21	2	2	0	0.145
77.12	3	1	1	0.124
**FAB NPs**				
29.88	2	2	0	0.298
35.23	3	1	1	0.255
37.91	1	1	1	0.237
43.69	4	0	0	0.207
46.11	2	0	0	0.197
57.02	5	1	1	0.161
62.63	4	4	0	0.149
64.21	2	2	0	0.145
77.12	3	1	1	0.124
